# Late onset of unilateral optic disk edema secondary to treatment with imatinib mesylate

**DOI:** 10.1002/ccr3.1137

**Published:** 2017-08-15

**Authors:** Mariasanta Napolitano, Marco Santoro, Salvatrice Mancuso, Melania Carlisi, Simona Raso, Giuseppe Tarantino, Vincenzo Accurso, Sergio Siragusa

**Affiliations:** ^1^ Hematology Division University Hospital Policlinico “Paolo Giaccone” via del Vespro 129 Palermo 90127 Italy

**Keywords:** Adverse events, chronic myeloid leukemia, imatinib, optic disk edema, optic nerve edema, tyrosine kinase inhibitors

## Abstract

Prompt ophthalmology evaluation and immediate imatinib suspension should be suggested at any time of tyrosine kinase inhibitor therapy in patients with visual deficit, as it may be a clinical manifestation of optic disk edema, and suspension may help in prompt recovery.

## Introduction

Imatinib Mesylate is a tyrosine kinase inhibitor (TKI) indicated for the treatment of chronic myeloid leukemia (CML), acute lymphoblastic leukemia and gastrointestinal stromal tumors 1, 2, 3. Imatinib and other TKIs act by inhibiting the function of the BCR‐ABL chimeric molecule and have dramatically improved the prognosis of these diseases since their approval. Known ocular adverse events (AEs) related to imatinib treatment are periorbital swelling, epiphora, blepharoconjunctivitis, extraocular muscle palsy, and palpebral ptosis 1, 4, 5, 6, 7. Edema is a known common AE related to imatinib treatment and usually presents as Common Terminology Criteria – AE grade I–II, peripheral lower limbs, and/or periorbital edema. Retinal edema and cases of optic disk edema during imatinib treatment are anecdotally reported, with usual onset during the first 3–6 months of therapy 7, 8, 9.

Kwon et al. 8 reported the case of bilateral optic disk edema causing photopsia and visus loss in a 14‐year‐old Korean female diagnosed with CML and treated with imatinib for 2 months. The symptoms improved after 2 weeks of TKI discontinuation, and imatinib was started again a week later. No other ocular symptoms appeared since then 8. Kusumi et al. 7 reported the occurrence of retinal edema after 6 months of imatinib treatment on a 31‐year‐old female, complaining of visual deficit. The AE improved 2 weeks after the suspension of imatinib and completely resolved on the 32nd day of off‐therapy period 7. De Luca et al. 9 reported a case of unilateral painless loss of vision in the left eye of a patient in which imatinib had been increased from 200 mg a day to 400 mg a day 3 months before 9.

We here report the case of a patient with CML who developed unilateral optic nerve edema after more than 9 years of imatinib treatment.

## Description

A 62‐year‐old Caucasian male was diagnosed with CML according to WHO criteria in March 2007. He was treated with imatinib (400 mg a day) since that date and achieved optimal response at the 3rd and 6th month according to ELN criteria 10. At the time of diagnosis, the patient did not report any comorbidity (in particular not diabetes nor arterial hypertension) and had optimal performance status with ECOG‐PS 0, according to the Eastern Cooperative Group score. He had not been taking any concomitant drug therapy when he started imatinib treatment.

In August 2015, he was diagnosed with acute pancreatitis and imatinib was suspended for almost a month until complete recovery, without losing MMR. In September 2015, neither symptoms nor signs of pancreatic disease at pancreatic and liver function examinations and imaging (cholangio‐MRI) were reported. Imatinib treatment was therefore started again at a lower dose and then gradually increased to 400 mg a day.

**Figure 1:**
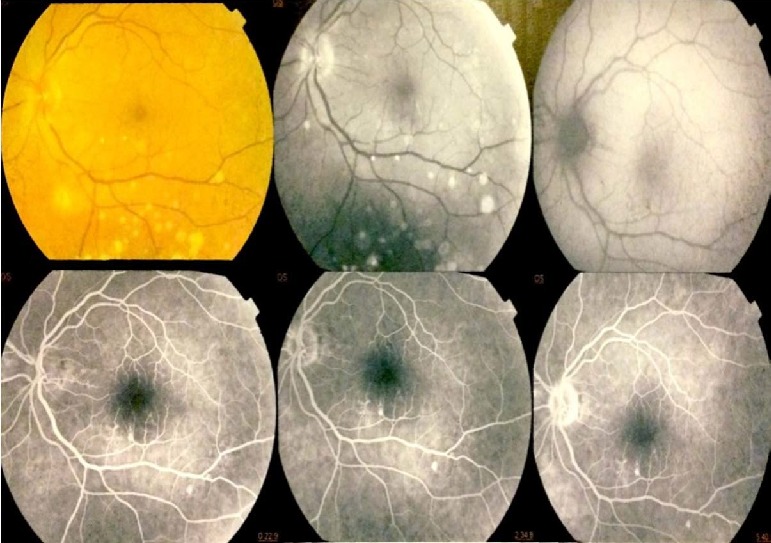
Fundus oculi at the time of papilloedema diagnosis.

On July 2016, the previously normal‐sighted patient referred to an ophthalmologist for visual alterations – slow onset visus deficit and gravative acute and transient ocular pain – in his right eye. A fundus campimetry revealed inferior and superior arcuate scotoma affecting the right eye (Fig. 1). Subsequent fluoangiography excluded thrombotic events and optic coherent tomography revealed signs of optic disk edema of the right eye, without any alteration in the left eye. In order to rule out intracranial masses or other possible causes of unilateral optic nerve edema, he underwent contrasted cranium computed tomography and magnetic resonance that resulted unremarkable. The patient did not complain of any other symptom. At clinical examination, no peripheral edema and/or other clinical sign of note was revealed. At the moment of the AE, the response to TKI therapy was major molecular response (MMR) with an International Standard Peripheral Blood BCR‐ABL1/ABL1 of 0.012 (MR3) on polymerase chain reaction (PCR). According to the literature, imatinib was therefore interrupted and a hematologic monthly follow‐up was planned. The patient underwent neurologic and infectious disease specialist evaluation that ruled out infective causes. Lumbar puncture was not performed, as the patient reported visual improvement and a new ophthalmological evaluation revealed an objective reduction of the optic disk swelling after the first week of imatinib suspension. Ocular transient pain was not explained. On August 2016, a control peripheral blood PCR revealed stability of MR3, with BCR‐ABL1/ABL1 IS = 0.061 and the patient announced sensible improvement of the visus alteration and complete remission of ocular pain, with an almost complete function recovery. We decided to continue the TKI withdrawal, also relying on the stability of the MMR. On September 2016, the ocular deficit was almost completely recovered, but MR3 was lost (BCR‐ABL1/ABL1 IS = 0.8). Therefore, a second‐line treatment with second‐generation TKI nilotinib has been started. A residual myopic deficiency needed correction with lenses, but it cannot be excluded it was already present before the onset of AE, because the patient did not carry lenses before the onset and had not underwent a recent ophthalmologic sight evaluation. At the last available PCR control, MMR was detected; neither systemic nor specific symptom was recorded at the clinical follow‐up.

## Discussion

Most common known ocular AEs secondary to imatinib treatment are periorbital edema, epiphora, conjunctival hemorrhage, blepharoconjunctivitis, visus alterations, and ocular dryness. Retinal edema is defined as a rare known ocular AE related to imatinib 1, 6, 7, 8. According to the literature, these AEs usually occur in the initial phases of treatment 7, 8, 9

Fluid retention commonly occurs during imatinib and other TKIs treatment and it may lead to diverse AEs, according to the target tissue. Edema has been related to the inhibition of platelet‐derived growth factor receptor (PDGFR) 7. PDGFR is involved in fluid regulation, and PDGFR *knockout* mice have been demonstrated to lack microvascular pericytes, which are part of the normal capillary wall 10, 11. Retinal edema may occur during TKIs treatment, rarely though, as the retina is a PDGFR‐expressing tissue.

In our case, retinal edema appeared 9 years after starting imatinib, but promptly resolved after drug suspension, without any specific treatment. The correlation between the AE and the drug is supported by dechallenge criterion: the grade of the AE improved after the suspension of imatinib.

Unilateral optic disk edema may be associated to high intracranial pressure, but more often is secondary to intracranial masses, cerebral sinus thrombosis, or optic neuritis 8. In our case, none of these complications were detected by imaging. Treatment discontinuation led to a gradual and almost complete regression of the ocular symptoms. Stable molecular response, according to the peripheral blood PCR, was maintained for the first 2 months after the imatinib cessation. Although a sustained resolution of AEs was reported even after the resumption of imatinib, we preferred to maintain the TKI suspension along with a monthly MR detection. Neither clinical signs nor symptoms suggesting a disease progression have been detected, but MMR was lost and nilotinib was started as second line of therapy.

In conclusion, we highlight the occurrence of optic disk edema due to imatinib even after a very long period of treatment and in the absence of cerebral masses, thrombotic, or infective events. Prompt ophthalmology evaluation and immediate imatinib suspension, even before obtaining specific diagnosis, should be suggested at any time of imatinib treatment in patients referring sudden visual deficit.

## Authorship

MN: performed writing and text review. MS: performed writing and references collection. SM: performed data collection and text review. MC and SR: performed literature review. GT and SS: performed text review. VA: performed writing and data collection.

## Conflict of Interest

None declared.
